# Autovaccination revisited: potential to boost antiviral immunity and facilitate HIV-1 cure/remission in children

**DOI:** 10.1097/COH.0000000000000924

**Published:** 2025-03-19

**Authors:** Harriet R. Parker, Julia E. Edgar, Philip J.R. Goulder

**Affiliations:** a Peter Medawar Building for Pathogen Research, Department of Paediatrics; b Peter Medawar Building for Pathogen Research, Nuffield Department of Medicine, University of Oxford, Oxford, UK; c Ragon Institute of Massachusetts General Hospital, Massachusetts Institute of Technology and Harvard University, Cambridge, Massachusetts, USA; d Africa Health Research Institute, Durban, South Africa

**Keywords:** autovaccination, CD8^+^ T-cells, early ART initiation, HIV cure/remission, paediatric HIV

## Abstract

**Purpose of review:**

To review the concept of autovaccination as a strategy to boost anti-HIV-1 immunity and improve immune control, especially as a means to facilitate cure/remission in paediatric HIV-1 infection, where effective interventions in clinical testing remain limited compared to adults.

**Recent findings:**

Early autovaccination studies, conducted 15–25 years ago, suggested potential immunological benefits from exposure to autologous virus in both children and adults, specifically when antiretroviral therapy (ART) was initiated during acute infection. More recent work in nonhuman primates (NHPs) has shown that early ART initiation can significantly reduce the viral setpoint following treatment interruption, primarily through CD8^+^ T-cell responses, and prevent early immune escape - a phenomenon commonly observed in ART-naive acute infections. Additionally, NHP studies indicate that multiple, short analytical treatment interruptions (ATIs) can delay viral rebound and further lower the viral setpoint via enhanced CD8^+^ T-cell responses.

**Summary:**

Recent studies in NHP support the potential for autovaccination via short ATIs to enhance antiviral immunity and improve immune control of HIV-1. With well tolerated, well monitored ATI protocols, autovaccination could be a valuable approach to facilitating cure/remission in children living with HIV (LWH), in whom very early-ART initiation and early-life immunity are associated with low viral reservoirs and high cure/remission potential.

## INTRODUCTION: RATIONALE FOR AUTOVACCINATION IN CHILDREN LIVING WITH HIV

At the turn of the millennium, the advent of effective antiretroviral therapy (ART) led to studies suggesting that early ART initiation followed by structured treatment interruption (STI) could improve immune control against HIV-1. It was hypothesized that autovaccination, defined as the short-term exposure to autologous virus, sufficient to boost HIV-specific immunity but not to precipitate immune escape and exhaustion, could facilitate immune control. Ultimately, this might enable people living with HIV-1 (PLWH) to maintain ART-free aviraemia and normal-for-age health. Early studies examining the impact of STIs had mixed results. Concurrently, there were significant advances in ART to once-daily, singlet-tablet combination regimens with minimal side effects, therefore weakening the rationale for further STI studies. However, as described below, the anticipated success of ART for children in low and middle-income countries has fallen short of expectations. The need for strategies to achieve HIV-1 cure has necessitated analytical treatment interruption (ATI) studies to assess novel treatment strategies. These studies have renewed interest in understanding the effects of treatment interruption on the immune response to autologous virus and the potential role of autovaccination in facilitating HIV-1 remission. Evaluating the impact of autovaccination is particularly relevant for children LWH, where curative intervention options under clinical investigation are more limited compared to adults.

There are currently an estimated 39 million people living with HIV-1 globally [1]. While ART has redefined the epidemic, mothers and children are left behind in terms of treatment. In sub-Saharan Africa, women and girls accounted for 62% of total new HIV-1 infections in the region in 2023 [1]. Furthermore, nearly 90% of the world's children LWH are concentrated in sub-Saharan Africa and only a subset of these children on ART maintain complete aviraemia - even in research settings [2,3,4^▪^,^5^]. Both children and adolescents face major challenges in achieving optimal ART adherence [6,7]. As life-long ART is currently the only treatment option available, alternative strategies are needed for children LWH to improve their health outcomes. Therefore, there is a need to explore strategies to achieving HIV-1 cure/remission.

Paradoxically, despite significantly worse HIV outcomes reported in ART-naive children compared to ART-naive adults [8,9], the potential for very early ART-treated children to achieve HIV-1 remission may be higher than in adults [10,11]. First, ART can be initiated immediately after birth, shortly following in-utero transmission, minimizing the time of untreated infection. Additionally, in the majority of cases, mothers receive ART prior to delivery, resulting in infants being exposed to treatment through placental transfer even before birth [4^▪^,^12^]. Second, the early-life immune system is naïve and highly tolerogenic, leading to a decreased frequency of activated memory CD4^+^ T-cells present for the virus to infect [13]. Third, vertically transmitted virus tends to have a low viral replicative capacity, which greatly impacts disease progression [4^▪^,^14^]. Collectively, these factors limit the size of the early-life viral reservoir [15,16], thereby increasing the potential for HIV-1 remission or cure in children LWH. 

**Box 1:**
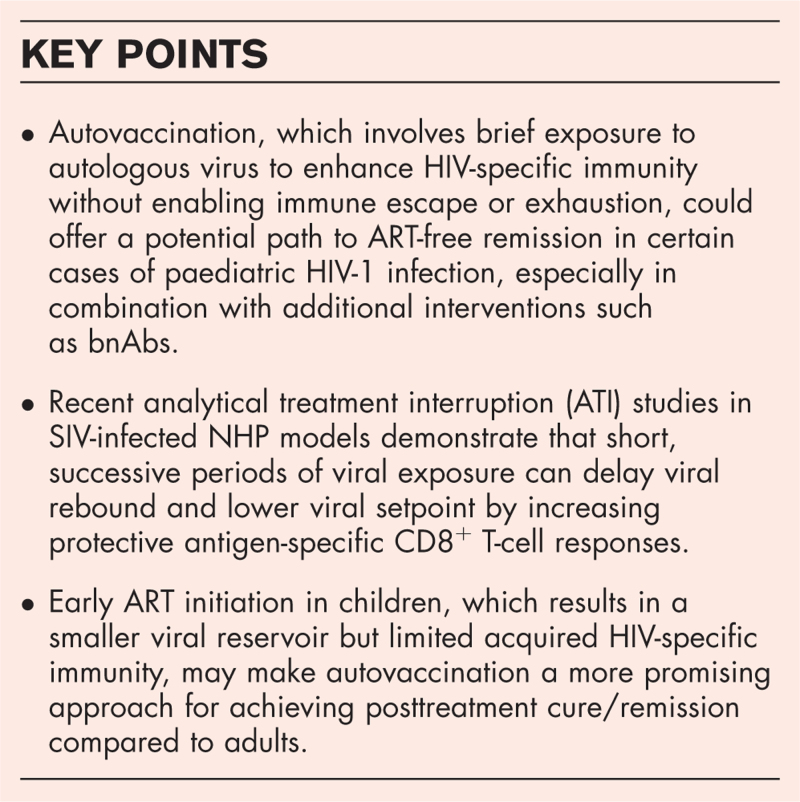
no caption available

**FIGURE 1:**
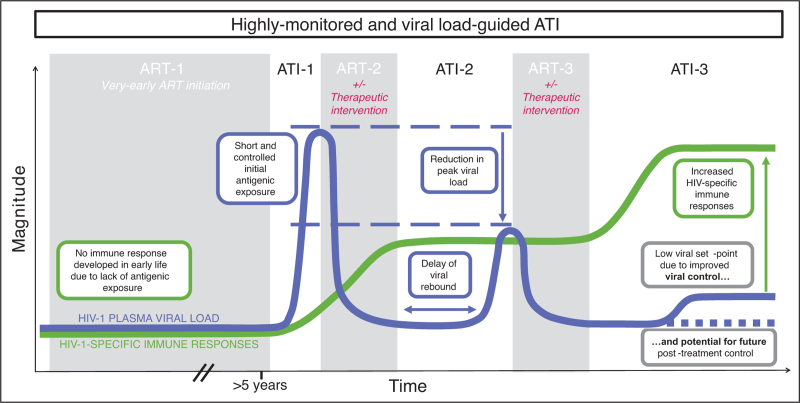
Schematic of potential HIV-1 plasma viral load and immune response dynamics in a pediatric multiple-ATI study evaluating autovaccination. Early-ART treated children LWH with sustained aviremia may undergo an initial ATI (ATI-1) to prime HIV-specific immune responses, followed by ART reinitiation immediately after viral rebound detection. Once viral suppression is re-established, a second ATI (ATI-2) may show delayed rebound and lower viral peak due to immune activation elicited during ATI-1. Additional ART/ATI cycles, with or without other therapies, could further lower viral set-point or, in some cases, achieve remission/cure. ART, antiretroviral therapy; ATI, analytical treatment interruption; LWH, living with HIV.

An unintended consequence of very-early ART initiation and the tight regulation of pro-inflammatory responses in early-life immunity is that, in those children with the smallest viral reservoirs and who are most likely to achieve HIV-1 cure/remission, de-novo anti-HIV-1 antibody responses and HIV-specific T-cell responses are often either limited or undetectable [17–20]. In these cases, an unscheduled treatment interruption would likely lead to unrestrained high viremia, rapid selection of escape mutations in CTL epitopes that mediate partial viral control, and immune exhaustion. Instead, short viral exposure through monitored ATIs may promote autovaccinational effects, utilizing the autologous virus to prime specific immune responses. With the ongoing challenges of developing HIV-1 vaccines, particularly in overcoming the extreme viral diversity both globally and within-individuals [21], autovaccination may offer a personalized alternative. It is likely that two or more ATI cycles may be required, as during an initial ATI, no adaptive anti-HIV-1 responses may be present to contribute to immune control of the rebounding virus. The initial ATI (ATI-1) serves two purposes (Fig. 1): first, to determine whether cure/remission has already been achieved, as has been described in a small minority of very early ART-treated children [4^▪^,^22^–^25^]; and, second, in children in whom virus does rebound, to boost HIV-1-specific immunity. To minimize viral escape and immune exhaustion, ART resumption should include virological criteria [26^▪^,^27^]. The exposure to viraemia would optimally be as brief as possible, bearing in mind that activation of the viral reservoir precedes detectable viraemia. After ART has been resumed for a period, and HIV-1 DNA in peripheral blood reaches pre-ATI levels of undetectability again, a second ATI (ATI-2) may be initiated. In theory, the presence of preexisting antiviral immunity will contribute to a delay in viral rebound and improved immune control if an appropriately monitored viral setpoint is allowed during this and any subsequent ATIs.

This autovaccination approach, mediated by one or more ATIs, could potentially provide long-lasting immune protection, enabling posttreatment control for certain children even without additional therapeutics such as broadly neutralizing antibodies (bnAbs), immunostimulatory agents (e.g., TLR7/9 agonists, type-I interferon stimulation, IL-15 superagonists), and latency reversal agents (e.g., histone deacetylase inhibitors). In recent years, ATIs have been used primarily as a tool to evaluate the efficacy of these interventions in adults. However, for immunotherapies such as bnAbs, which are reported to induce humoral and cellular responses that enhance viral control beyond neutralization [28–30], the necessary inclusion of ATIs in clinical trials to assess their efficacy complicates efforts to determine the specific contribution of each intervention. To advance toward an HIV-1 cure, it will be important to differentiate between favourable baseline participant characteristics, protective immune responses driven by autovaccination during treatment interruption, and the vaccinal effects directly elicited by therapeutics, while also exploring how these mechanisms can be optimized to work synergistically.

In this review, we evaluate how the autovaccination hypothesis has been tested in nonhuman primate models (NHPs) and in humans and outline the potential role autovaccination might play in facilitating HIV-1 cure/remission in children.

## EARLY STUDIES OF AUTOVACCINATION

In 1999, the Berlin patient was the first case study to report posttreatment control [31]. After an initial interruption where viremia rebounded, a second interruption saw no viral rebound and viraemia was suppressed in a 2-year ART-free follow-up. No HIV-specific neutralizing antibody responses were detected, but broad Gag-specific CD4^+^ and CD8^+^ T-cell responses were observed. This report provided the rationale for further investigations into ATI and the autovaccination effect.

Following this, several small studies demonstrated that early-ART-treated participants achieved ART-free immune control following multiple STIs, associated with increased virus-specific immune responses [32–34]. At the same time, studies of STIs in individuals who initiated ART during chronic infection suggested that this approach did not facilitate improved immune control [34–39], with the exception of one study [40]. In addition, the SMART study, with over 5000 participants who initiated ART in chronic infection, demonstrated that an STI design involving CD4^+^ cell count guided ART resumption increased the risk of opportunistic disease or death and major cardiovascular, renal, or hepatic disease [41]. A systematic review of treatment interruption studies published during the 2000 s era noted that this period often used longer treatment interruption durations, more lenient resumption criteria (time-based, CD4^+^ cell count guided, or plasma viral load-guided), and less frequent monitoring than is standard now [42,43].

In contrast with these concerning findings from treatment interruption studies in adults who initiated ART in chronic infection, the three published paediatric treatment interruption trials reported in children showed that closely monitored ATIs were well tolerated (reviewed by 32; [44–46]). These trials undertaken in the 2000 s used CD4^+^ cell count guided ART resumption and included children as young as 12 months of age. Long-term follow up of study participants also showed no detrimental impact of treatment interruption on cognitive function or neurological development [47–50].

The first evaluation of the autovaccinational effects of treatment interruption in children was conducted by Borkowsky *et al.*[51] using a repeated short-cycle ATI model. In a subset of participants (median age: 7 years), after a median of 14 ART/ATI cycles, IFN-g ELISpot responses to HIV-1 peptide pools increased six-fold and was associated with a decline in plasma viral load. Similarly, in a smaller study of four children (median age: 10 years) who underwent 3 ART/ATI cycles, in which immune responses were not measured, median viral load declined by 0.85 log_10_ between the first and third ATI [52].

In summary, the small number of paediatric ATI studies that were conducted in this era were well tolerated, and in the limited instances where plasma viral load and immune responses were assessed, findings consistently indicate a beneficial effect of ATIs on reducing plasma viral load and increasing HIV-specific T-cell responses. As evidence continues to emerge, it will be important to continually examine the balance of the benefits and risks for participants.

## AUTOVACCINATION STUDIES IN NON-HUMAN PRIMATE MODELS

Recent treatment interruption studies in SIV-infected NHPs have highlighted the central role of priming antigen-specific CD8^+^ T-cell responses in facilitating immune control of immunodeficiency virus infection, with or without additional therapeutics. In an early study by Lori *et al.*[53], rhesus macaques experimentally infected with the viral swarm, SIVmac251, underwent three ART/ATIs cycles, each comprising of 3 weeks on therapy followed by 3 weeks off, before a final setpoint ATI. With each successive cycle, viraemia declined, ultimately leading to complete immune control of SIV in 6/6 animals. This control was associated with enhanced SIV-specific CD8^+^ T-cell activity. In contrast, a single, prolonged treatment interruption followed by a setpoint ATI failed to achieve sustained immune control in any of six animals [53].

More recently, Okoye *et al.*[54] observed that rhesus macaques infected with the viral clone, SIVmac239, who initiated ART 12 days postinfection and remained on treatment for 12 months before ATI, had a mean viral setpoint of approximately 4.5 log_10_ copies/ml (c/ml). This setpoint was approximately 2 log_10_ lower than that reported in rhesus macaques infected with SIVmac239 without ART administration [54,55]. Notably, depletion of CD8^+^ T-cells by anti-CD8 mAbs reversed this 2-log reduction in setpoint [54]. These findings indicated that early ART might improve the effectiveness of virus-specific CD8^+^ T-cell responses, potentially by preventing early viral escape, thereby enabling an effective CD8^+^ T-cell response against the rebounding virus during ATI compared to that seen in acute ART-naive infection. This notion is supported by the study by Docken *et al.*[56^▪▪^], in which rhesus macaques infected with SIVmac239 began a 30-week course on ART 14 days postinfection, similarly resulting in a reduction of median viral setpoint to 3.62 log_10_ c/ml during the first ATI. It was shown that the initial period on ART had prevented viral escape from the dominant Tat-SL8-specific CD8^+^ T-cell response in Mamu-A∗01 animals. Indeed, following early ART, the frequency of the Tat-SL8-specific CD8^+^ T-cells increased almost eight-fold over the ensuing 30 weeks on therapy. By contrast, in ART-naive Mamu-A∗01 animals, viral escape mutants from the Tat-SL8 response emerge as early as 2 weeks postinfection and reach fixation by approximately 4 weeks postinfection [55]. Consistent with the study by Lori *et al.*[56^▪▪^], a second cycle of treatment followed by an ATI resulted in a delayed time to viral rebound, reduced mean viral load peak, and further lowering of the viral setpoint to 2.2 log_10_ c/ml. This viral setpoint is therefore 4.3 log_10_ lower than reported in similarly infected ART-naive animals [55]. For many ART-naive people LWH, a 4.3 log_10_ reduction in median viral setpoint would likely result in a setpoint below the limit of detection in standard assays of plasma viral load (20-30 HIV-1 RNA c/ml) [57]. Once again, in these rhesus macaques studies, the reductions in viral setpoint were largely reversed by CD8^+^ T-cell depletion using anti-CD8 mAbs [56^▪▪^].

Interestingly, a study evaluating ART initiation timing in NHPs revealed that *delaying* ART up to three weeks postinfection was associated with *lower* viral peak and viral set point during ATI, while further ART delays worsened outcomes [58]. These findings are reminiscent of the RV411 study in which ART was initiated in Fiebig I (ie prepeak viraemia in acute infection [59]) and continued for a median of 2.8 years prior to ATI [60]. All eight participants in this study experienced very rapid viral rebound, after a median of 26 days’ ATI [60]. By contrast, the median time of ART initiation in the VISCONTI cohort of posttreatment controllers was in Fiebig V (approximately 3 months after infection [59]) [61]. These findings together suggest that, if ART is initiated too early, the immune system may not be sufficiently primed to enable effective antiviral immunity in the event of a subsequent ATI. On the other hand, if ART is initiated too late, immune escape and exhaustion result and, again, inadequate antiviral immunity is accessible to facilitate immune control when treatment is interrupted [62].

Studies of other HIV therapeutic approaches in NHPs, such as bnAbs, further illustrate the importance of antigenic exposure in inducing effective CD8^+^ T-cell responses. SHIV-infected rhesus macaques treated with bnAbs 10-1074 and 3BNC117 shortly after infection showed delayed viral rebound compared to continuously ART-treated animals, with 6/13 achieving long-term control even after bnAb concentrations declined below therapeutic levels [26^▪^]. CD8^+^ T-cell depletion in these controllers led to rapid viral rebound, consistent with their central role in maintaining immune suppression of viraemia [63,64]. The authors speculated that, unlike continuously ART-treated controls, bnAb treatment may sustain low-level viremia, promoting immune complex formation with viral antigen that engages Fcγ receptors (FcγRs) and enhancing antigen-presenting cell-mediated activation of T-cells, among other effector functions [65,66]. This mechanism is referred to as the vaccinal effect and is potentially key to achieving long-lasting suppression [29,67]. Indeed, it has been reported previously in humanized mice [68,69] and in NHP models that optimized FcγR-binding bnAbs improve viral control [70,71] and can induce higher frequencies of IFNγ-producing CD8^+^ T-cells compared to Fc-null bnAb variants [72]. Notably, SHIV-infected rhesus macaques administered 10-1074 and 3BNC117 mAbs alongside N-803, an IL-15 superagonist that elicited transient viremia while on ART, resulted in increased CD8^+^ T-cell function and sustained CD8^+^ T-cell dependent viral control during ART interruption [73^▪^]. Collectively, these findings from NHP models suggest that CD8^+^ T-cells, primed during acute infection or low-level antigenic exposure with or without other immunotherapies, are important to achieving durable viral suppression.

## AUTOVACCINATION APPROACHES TO FACILITATE PAEDIATRIC CURE

As mentioned above, the increased feasibility of initiating ART very early in paediatric infection, combined with the tolerogenic nature of early-life immunity, offer a significant opportunity to minimize the size of the viral reservoir. The downside of these factors is that HIV-specific immunity is virtually absent following early ART. In those children who have maintained ART-mediated suppression of viraemia until the time of ATI, there is virtually no effective adaptive anti-HIV immunity available to assist with immune control. Therefore, implementing a protocol comprising two or more ATIs could enable the paediatric immune system to benefit from the induction and/or boosting of antiviral adaptive immunity during a short initial ATI, followed by a set-point ATI in which these HIV-specific responses can contribute to immune control (Fig. 1). This approach is also likely to benefit children who subsequently undergo unscheduled treatment interruptions, as is common in paediatric and adolescent settings, in that a preexisting broad antiviral adaptive immune response would be on hand to contribute to effective immune control in such a contingency. As previously described, the options for additional interventions to help achieve cure currently in clinical testing are substantially more limited in children than adults. However, if bnAbs are available, employing these in combination with ART at the time of ART resumption following viral rebound ensures that these are given in the setting of viraemia, which arguably may maximize their ability to form immune complexes and mediate vaccinal effects. This strategy has been used effectively in adult bnAb trials such as the eCLEAR study and is also a part of the study design for the ongoing RIO trial [74,75]. Furthermore, the use of ATI in well designed paediatric trials will offer an opportunity to identify viral and host immune factors associated with enhanced ART-free viral control which can be used to optimize and increase access to effective interventions for children LWH [26^▪^].

A final important consideration is the optimal timing of autovaccination and other interventions designed to facilitate cure/remission in children LWH. Immune ontogeny does not support high-frequency, broad and multifunctional HIV-specific CD8^+^ T-cell responses in early life [76–78]. Particularly in the first 2 years after birth, virus-specific CD8^+^ T-cell responses are relatively ineffective [79]. However, at 5 years of age or older, these responses start to have a significant impact on containing viral replication [80]. Additionally, current consensus guidelines advise against ATIs in children under 2 years of age due to the neurodevelopmental risks associated with viraemia exposure during this critical period [26^▪^,^27^]. However, as children grow into adolescents, this brings about many other psychosocial challenges associated with this demographic, including treatment adherence, access and retention of care, and navigating sexual relationships [81–83]. Thus, the optimal age to initiate autovaccination and other interventions designed to facilitate cure/remission in very early ART-treated children may be from 5 to 10 years of age. Future research will need to evaluate the optimal design and efficacy of autovaccination strategies in children to maximize immune benefits while minimizing adverse risks.

## CONCLUSION

Autovaccination, elicited by monitored ART interruption, presents a potential strategy to enhance immune control and facilitate HIV-1 remission in certain children. While very early ART initiation and a tolerogenic immune environment may restrict the size of the viral reservoir in young children, it additionally prevents the development of protective HIV-specific immune responses. Currently, with no approved therapeutic options beyond ART, particularly in resource-limited settings where ART adherence and viral suppression are often suboptimal, children require alternative well tolerated and effective strategies to achieve HIV-1 cure/remission. Evidence from both human and NHP studies suggest that controlled antigen exposure via short ATIs can strengthen HIV-specific T-cell immunity, delay viral rebound, and reduce the viral setpoint, possibly even enabling long-term ART-free suppression. As the development of other immunotherapies continue to progress, incorporating interventions such as bnAbs alongside ATIs may further enhance their protective modulation of host immunity. Importantly, inclusion of ATIs will additionally allow for the identification of viral and host immune factors that are associated with ART-free viral control in children LWH, aiding the development of improved therapeutics for paediatric HIV-1. Further investigation using modern ATI protocols with frequent monitoring is needed to better define the impact of autovaccination in generating sustained, durable anti-HIV immune responses and ultimately achieving cure/remission.

## Acknowledgements

*None.*

### Financial support and sponsorship

*This work is supported by the National Institutes of Health (PG UM1-AI164566 and UO1AI168655).*

### Conflicts of interest

*None.*

## REFERENCES AND RECOMMENDED READING

Papers of particular interest, published within the annual period of review, have been highlighted as:

▪ of special interest
▪▪ of outstanding interest
